# Inside “Pandora’s Box” of Solidarity: Conflicts Between Paid Staff and Volunteers in the Non-profit Sector

**DOI:** 10.3389/fpsyg.2020.00556

**Published:** 2020-05-04

**Authors:** Rocío López-Cabrera, Alicia Arenas, Francisco J. Medina, Martin Euwema, Lourdes Munduate

**Affiliations:** ^1^Department of Social Psychology, University of Seville, Seville, Spain; ^2^Occupational & Organizational Psychology and Professional Learning, Katholieke Universiteit Leuven, Leuven, Belgium

**Keywords:** non-profit organizations, paid staff, volunteers, organizational conflicts, negative emotional consequences

## Abstract

Non-profit organizations (NPOs) are quite complex in terms of organizational structure, diversity at the workplace, as well as motivational mechanisms and value rationality. Nevertheless, from the perspective of organizational psychology, the systematic analysis of this context is scarce in the literature, particularly regarding conflicts. This qualitative study analyzes types, prevalence, and consequences of conflicts in a large NPO considering as theoretical framework several consolidated organizational psychology theories: conflict theory, social comparison theory, and equity theory. Conflicts were analyzed taking into account volunteers’ perspective, who have been the consistent protagonist in NPO research, but also considering paid staff’s perspective as one of the main stakeholders in these organizations, whose relative power has increased in the past decade due to the professionalization of the NPO’s sector. Results confirmed the existence of four types of conflicts: task, process, status, and relationship conflicts. Relationship conflict is the least reported type, revealing the protection factor that values and engagement with a social aim have on this organizational context. The most relevant finding is the strong difference between paid staff and volunteers in conflict perceptions, showing paid staff, overall, higher levels of conflicts than volunteers. Findings also show stronger negative consequences for paid staff compared to volunteers. Theoretical and practical implications are discussed.

## Introduction And Theoretical Background

The third sector in economy is used to define different kinds of organizations (e.g., non-profit organizations – NPOs – or charities) that do not fit in neither the public nor private sector (Corry, 2010). During the past decades, the third sector has acquired special relevance in areas such as employment wealth and social welfare (Cabra de Luna, 2016). Based on its ambivalence or “hybridity” (Brandsen et al., 2005), these organizations are able to look out for social needs that neither public nor private institutions are able to fully satisfy, the former due to their difficulties to prevent and control these needs, and the latter due to their focus on profits (Corral-Lage et al., 2019). Third sector organizations not only demonstrated their resilience, showing a minor decrease on their unemployment rate during the last economic crisis (CIRIEC, 2012), but also promoted the personal well-being of those who lost their job by means of volunteering or participating in training programs (Kamerāde, 2015). This role was especially outstanding in countries that suffered a major impact during that period, such as Spain. In these countries, despite their own difficult financial situation, third sector organizations assumed a subsidiary role as opposed to the institutional role of the State, guaranteeing the social welfare and vulnerable groups’ rights.

Third sector organizations are characterized not only by working toward a mission based on common values and commitment (Etzioni, 1973) but also by their institutional character, non-profit distribution, self-governing, and volunteerism (Salomon and Anheier, 1997). In the third sector, NPOs especially stand out for their diversity and heterogeneity of goals (Alcock and Kendall, 2011) as well as their organizational complexity in terms of motivational mechanisms, value rationality, and organizational structure (DiMaggio and Anheier, 1990; Will et al., 2018). Indeed, these distinctive features, together with the relevance that these organizations acquired during the last decades, turned NPOs into a growing research topic.

Among all, a main characteristic of NPOs is the coexistence of paid staff and volunteers as part of the same working teams (Corry, 2010). This NPO’s distinctive trait is, at the same time, the source of mutual enrichment that contributes to achieving their social and organizational goals and a source of confrontation between these groups (Kreutzer and Jäger, 2011). One of the clearest examples of this dichotomy is the growing trend of “professionalism” in these organizations. This “professionalization,” as a response to financial needs, directly clashes with volunteerism and prosocial ideals (Maier et al., 2016). Consequently, not only their social labor and effectiveness can be affected by potential conflicts but also their own job satisfaction and motivation (Netting et al., 2008). These prospective negative consequences justify further research on conflict in NPOs.

In this regard, previous literature on this topic highlights the existence of high levels of conflict between these actors in NPOs (Pearce, 1993; Netting et al., 2008; Macduff, 2012), however, the mechanisms underlying these circumstances are still unclear. Even though both stakeholders proved to be essential to maintain NPO activity, there is still a lack of awareness about how and why conflicts develop in this organizational context and which are the specific conflicts that take place between these actors. Indeed, those conflicts are not clearly defined in the non-profit research based on established conflict typologies, so their prevention and management area challenge both parties and organizations. These conflicts have been described in terms of “difficulties” between volunteers and paid staff due to their different roles in the organization (Kreutzer and Jäger, 2011; McAllum, 2018). While the causes of these difficulties and the cognitive mechanisms involved have not been analyzed from the framework of grounded theories, their relevance and negative impact on job satisfaction have been widely confirmed (Netting et al., 2008). Overall, in light of the incidence of conflict, its consequences for individuals and organizations, and, above all, the lack of awareness about the mechanisms underlying these circumstances in NPOs, specific research on this matter is necessary and worth it.

The aim of this study is to make headway on the previous research findings analyzing conflicts in NPOs, contributing to their understanding, prevention, and management. Our research will be based on two relevant theoretical frameworks in organizational psychology: the conflict theory by Deutsch (1973) and the social comparison theory by Festinger (1954).

Deutsch (1973) explained that conflicts arise whenever incompatible activities or aspirations occur between parties, both in cooperative and competitive situations. According to this author, these situations can be triggered by (a) parties’ characteristics (their values and motivations, aspirations, and objectives); (b) their prior relations (including expectations); (c) the nature of the issue rising the conflict or the social environment in which the conflicts take place; (d) their strategy managing them; and (e) conflict consequences. It has been pointed out that in NPOs, conflicts arise particularly due to unclear boundaries between the main roles in the organization (McAllum, 2018), definitively two different and changing organizational identities with different ways of doing their jobs. This complex interaction between paid staff and volunteers will lead to different types of conflicts, depending on the nature of the issues at stake.

To understand how conflict arises between these two profiles, it may be helpful to rely on the principles of the theory of social comparison (Festinger, 1954). This theory states that individuals create their perceptions and opinions by means of comparison with other social groups. Thus, paid staff and volunteers also evaluate their contributions to work, rewards, and efforts based on those of the other main group in their working context. Considering the equity theory referring to motivational mechanisms proposed by Adams (1963, 1965), if there is no perception of fairness as a result of that social comparison, this unbalance can lead to conflicts between these two groups or identities, affecting their motivation and job satisfaction. Particularly in this context, we hypothesized that paid staff would perceive more conflict than volunteers due to a perceived negative balance between their contributions to work and the rewards they obtain.

Based on these premises of organizational psychology theories, we intend to improve the understanding of conflict in NPOs, particularly between the main stakeholders of these organizations, in order to provide the needed background to prevent and manage these situations that impairs both organizational members’ wellbeing and goals achievement. To do so, as specific objectives in this study, first, we will revise which types of conflicts can be found in NPOs, identified by paid staff and volunteers. Considering the actual roles of volunteers and paid staff in NPOs as well as social and contextual challenges – such as professionalism – that this sector is facing nowadays, we will analyze four types of conflict: task conflict, process conflict, status conflict, and relationship conflict (Jehn, 1995, 1997; Jehn and Mannix, 2001; Bendersky and Hays, 2012). Second, we will explore who reports more conflicts, paid staff or volunteers, and therefore, who experience more negative consequences. In this regard, paid staff will be especially attended as, compared to volunteers, research on their perceptions on NPO dynamics and conflicts is still scarce.

### Conflicts Between Paid Staff and Volunteers

As abovementioned, the relationship between the two main stakeholders (volunteers and staff) is nowadays an important topic of interest (Hwang and Suárez, 2019). To clearly understand the actual function of paid staff and volunteers as part of the same team in NPOs, Ariza-Montes et al. (2017) explain their different roles based on two criteria: professionalism and time available. Paid staff occupy responsible positions, overseeing complex activities due to their professional training, while volunteers collaborate on different tasks following a flexible schedule, based on their availability to participate and also their commitment (Ariza-Montes et al., 2015, 2017). However, paid staff’s and volunteers’ roles are sometimes not that easy to differentiate in NPOs. Their intricate organizational structure and the variety of tasks that they accomplish – sometimes considered incompatible activities – or their different aspirations not only make it difficult to clearly define their roles in the organization but also lead to disputes and conflicts among them (Deutsch, 1973). In fact, recent changes in the NPO sector, which imply more dependence on the external funding instead of on the NPO’s partner donations, have increased the need for professionalization to survive (Eisenberg and Eschenfelder, 2009). Therefore, the technical staff role is becoming more significant in this new NPO’s structure. However, despite its relevance, only some studies explored, and just in a descriptive manner, which are the conflicts that rise up in NPOs, as a consequence of paid staff’s and volunteers’ interaction. Indeed, it is not without a reason that Pearce (1993) described these conflicts as “one of the unpleasant secrets of non-profit organizations” (Pearce, 1993, p. 142). Further research is needed to clearly understand conflicts in NPOs.

In this regard, it is particularly remarkable how research has traditionally paid an unbalanced attention to both stakeholders: on the one hand, volunteers who usually capture the spotlight as protagonists of these organizations’ values and, on the other hand, paid staff whose needs, contributions, and role in NPOs’ dynamics are most of the times overlooked. As a matter of fact, recent studies deeply analyze the role of volunteers in NPOs, for instance, in terms of volunteers’ involvement, considering both organizations’ and the volunteers’ perspectives on commitment, intention to leave, or working conditions (Nesbit et al., 2017). Also, volunteers’ profiles, including their cultural and demographic correlates (Ariza-Montes et al., 2018), educational level, social resources, and volunteering consequences have been studied on NPO research (see Wilson, 2000 for a review). Even so, specific leadership strategies have been analyzed in order to guarantee an appropriate volunteering management (Studer, 2016). Nevertheless, research on NPOs has systematically overlooked a main actor’s perspective regarding NPO dynamics: paid staff’s perspective.

A possible explanation for these circumstances is that volunteering is one of the key NPOs’ social and human capital resources, understood as a proactive and committed behavior based on offering time freely to help others (Wilson, 2000) and providing a service to society (Farmer and Fedor, 2001). Cnaan et al. (1996) already highlighted that NPOs usually rely to a large extent on volunteers compared to paid staff. Therefore, previous studies mainly focused on the existence of volunteers, as a factor that determines both the organizational culture and identity of these organizations, where values, participation, and integration traditionally prevail over a managerial approach focused on effectiveness and efficiency (Kreutzer and Jäger, 2011).

This increasing interest resides on the importance that volunteers gained in order to maintain an optimal functioning of NPOs during difficult socioeconomic crisis periods (Baluch, 2012), particularly during financial constraints, both due to the absence of institutional support from public administration and the decrease in private donations (Salamon, 2010). Under these circumstances, NPOs were forced to recruit volunteers instead of hiring paid staff, blurring the lines between both roles. Consequently, some authors even consider that volunteers act sometimes as unpaid and low-skilled paid staff (Ganesh and McAllum, 2012). Thus, when both volunteers and paid staff evaluate their current situation in NPOs, considering the other role as a reference, it is likely that they feel their rewards are decreasing compared to the new demands imposed by the organization, particularly those related to work processes and power position (e.g., paid staff focusing on project management and volunteers assuming paperwork that keeps them far from face-to-face relationships with users) (Festinger, 1954). This comparison will contribute to the appearance of conflicts between these two stakeholders and, particularly, different types of conflict based on the issue that is raising the disagreement. Therefore, considering these NPO dynamics, paid staff’s and volunteers’ coexistence will contribute to the appearance of four types of conflict: process conflict, task conflict, status conflict, and relationship conflict.

These circumstances lead to the appearance of *process conflicts*, defined as disagreements about how a task should be accomplished, including issues such as who should do what and how much responsibility each member of the group should take (Jehn, 1997; Jehn and Mannix, 2001). Indeed, Mook et al. (2014) demonstrated that, on the one hand, 10.8% of volunteers reported that they replaced a paid staff member, with 3.1% of those cases permanently. On the other hand, volunteers also reported being replaced by paid staff: 7.6% reported being replaced, with 2.1% of those cases being permanently replaced.

However, during the last decade, paid staff are inevitably acquiring more prominence in NPOs due to their metamorphosis in business-like organizations, as a response to their context demands (Eisenberg and Eschenfelder, 2009; Maier et al., 2016). Governments’ policies, which nowadays rely on NPOs to provide basic social services to community (Henriksen et al., 2012), are setting proceedings to identify and finance the most effective NPOs (Salamon, 2015). This promotes the implementation of new procedures to maximize their productivity and adjust their methods to their applications’ requirements (Berzin and Camarena, 2018; McAllum, 2018). These administrative procedures usually must be assumed by paid staff, as they require a constant supervision that cannot be guaranteed by volunteers, who usually have a very flexible work schedule as most of them have a remunerated job position elsewhere (Ariza-Montes et al., 2015, 2017).

This new scenario leads to the appearance of *task conflicts*, which are defined as disagreements or different opinions about the contents of a certain task (De Wit et al., 2012). The same blurred boundaries between professionalization and volunteerism that lead to process conflict also provoke task conflict. NPOs’ professionalization does create discrepancies not only regarding who should accomplish each task (e.g., social assistance or administrative duties) but also concerning which task should be prioritized by the organization. Consequently, as Ganesh and McAllum (2012) explained, even volunteers are involved in administrative tasks at the expense of decreasing their time engaging with social issues due to professional restrictions.

To face their “professionalization” process, NPOs have adopted different measures as a transition method trying to maintain, to a greater or lesser extent, the rationale that would be expected from a volunteering-based organization. Thus, some NPOs transfer their direction to professionals or paid staff (e.g., Oxfam), while others maintain a traditional volunteer’s leadership (e.g., Mans Units). Some NPOs, on the contrary, assign formal and informal roles to paid staff and volunteers in an attempt to maintain a balance between financial needs and values: (a) a formal and institutional leadership role, where the NPO board is composed of volunteers who are in charge of governance and leadership, and (b) the informal or managerial level, where paid staff have the responsibility for the operations and daily management of the projects, and volunteers should execute complete tasks or substantial parts of the tasks. Under these circumstances, volunteers report difficulties to manage this professionalization process due to the complexity that entails to identify how these new demands and procedures can be balanced with a volunteering ideology (Kreutzer and Jäger, 2011). However, for paid staff, this change has also a considerable impact, since their job demands increase exponentially. They must deal with several administrative duties, performance management, and bureaucracy, tasks they often consider less relevant than their social labor, hindering their job (King, 2017).

Overall, the hierarchical structure of the NPOs, combining paid staff and volunteers, as well as the complexity of their work that requires a great coordination among organization members, seems to be an appropriate scenario to elicit also the so-called *status conflict*. This type of conflict implies trying to challenge or alter the implicit or explicit established hierarchy (Bendersky and Hays, 2012). The motives and base for participating in the organization might also drive volunteers to challenge the – often formal leading – role and position of paid staff. Also, paid staff might feel the need to reinforce their leading position to operational volunteers, while challenging the formal status of the board composed of volunteers with managerial responsibilities, who may have good intentions but not always agree with the new professionalism perspective adopted by NPOs (Kreutzer and Jäger, 2011).

Furthermore, previous research on this matter pointed out as a regular source of conflict the differences between volunteers and paid staff regarding which are their duties and responsibilities and their different criteria on how things should be done in the organization, for instance, in terms of volunteerism and professionalism clashes (Netting et al., 2008; Kreutzer and Jäger, 2011). Additionally, these disparities in criteria and even diversity among team members in the organization (e.g., in terms of backgrounds, age, and also roles) may even lead to a sense of identification in two different groups in the organization, paid staff and volunteers, that may justify a different perspective when it comes to described conflicts (Chenhall et al., 2016). This situation can also be a potential source of *relationship conflicts*, defined as the experience of personal incompatibilities or tensions that provoke feelings such as frustration or irritation (Jehn and Mannix, 2001). However, in NPOs, both volunteers and paid staff share a high commitment with their organization’s social labor, mission, values, and ideals (Brown and Yoshioka, 2003; Alfes et al., 2015). This commitment and common values can act as a barrier that prevents the appearance of this last type of conflict. Indeed, as the results of the study of Benitez et al. (2018) point out, using avoidance as conflict management strategy buffers the link between relationship conflict and negative emotional consequences in teams. Likewise, paid staff and volunteers may prevent relationship conflict appearance or escalation overlooking their personal differences to guarantee their projects’ success based on a common goal, that is, helping others in need. Moreover, the increasing job demands and hierarchical dynamics contribute to unbalanced paid staff’s and volunteers’ contributions–rewards ratio, and, as a consequence, not only they decrease their motivation but also, when they compare their situation with that of the volunteers, their perception of conflict increases, particularly for those types of conflict related with work processes (task and process conflict) and positions of power and influence (status conflict).

Therefore, considering the foregoing description of how paid staff’s and volunteers’ interaction and social comparison in the NPOs’ actual context can lead to different types of conflict, we propose that the following:

*Proposition 1*: (*a) Conflicts in NPOs can be categorized based on the taxonomy task, relationship, process, and status conflicts and (b) more task, process, and status conflicts will exist, as opposed to relationship conflict that, although existent, will be less prominent in this context.*

The heterogeneity of NPOs’ teams not only in terms of roles (paid staff and volunteers) but also in terms of background, age, or motivations can lead to disagreements regarding the content (task conflict) and who should conduct the tasks (process conflict). Given these circumstances, either paid staff or volunteers use their influence or positions to prevail or impose their perspectives or ideas as a reaction mechanism to face these organizational changes (status conflicts). Finally, such diversity is fertile soil for personal frictions between team members (relationship conflict); however, this last type of conflict may be mitigated due to both paid staff’s and volunteers’ commitment with their social labor and organizational values, who focus on a common goal instead of on their personal differences.

### Differences Between Paid Staff and Volunteers on Conflict Experience

As described before, previous research pointed out that the differences between volunteers and paid staff regarding which are their duties and responsibilities, as well as their different criteria on how things should be done in the organization, are regular sources of conflict (Netting et al., 2008; Kreutzer and Jäger, 2011). Indeed, as it was previously mentioned, these criteria’s disparities may even lead to a sense of identification in two different groups in the organization, paid staff and volunteers, that based on the social comparison theory (Festinger, 1954) and the equity theory (Adams, 1963, 1965) justify their different perspective when it comes to describing conflicts (Chenhall et al., 2016). Considering that the new trends in NPOs are increasing paid staff demands and responsibilities (e.g., professionalization; Maier et al., 2016), but their returns, in terms of recognition in the organization, salary, reputation, or sense of achievement, are still the same – or less – compared to volunteers, their perception of conflict and the negative effect of those perceptions on their satisfaction may be higher.

In order to evaluate how both paid staff and volunteers experience conflicts in NPOs, and therefore their consequences, context variables should also be taken into consideration. Thus, despite the large number of volunteers in these organizations (Cnaan et al., 1996), paid staff may be particularly vulnerable to experience conflicts in their teams. A main reason is that NPOs’ professionalization process would require also a change of the employees’ profile (Blake, 2012), encouraging an economic instead of social or vocational orientation (Piñón, 2010).

Nevertheless, paid staff profiles in NPOs are, on a normal basis, far away from these requirements, as they usually have a very prosocial background in terms of training (e.g., as health or human services professionals rather than as business professionals). However, instead of focusing their work on social intervention, paid staff have to, first, deal with volunteers who are in the board of their organization, to whom they have to report and, second, lead projects in which they depend on volunteers to do large parts of the job. In this matter, they must rely on the goodwill and commitment of these volunteers, experiencing a lack of formal power, even though they have responsibility and related authority (Medina et al., 2008).

These complex dynamics create internal conflicts between volunteers and paid staff (McAllum, 2018), being particularly detrimental for the latter, who are ultimately responsible for the technical implementation and administrative management of the different NPOs’ projects. Volunteers, on their behalf, feel less pressure to maintain their collaboration with a specific NPO in case they are not satisfied or they consider that their psychological contract with the organization has been breached or violated; since they are not bound by an employment contract, they feel free to contribute to their social causes somewhere else, leaving the organization and, therefore, avoiding negative conflict consequences to a greater extent compared to paid staff (Vantilborg, 2015). Overall, paid staff typically are “in-between,” usually working (more than) full time and in coordinating positions. Given their labor contract, they are also in different ways dependent on the organization. Therefore, their final contributions–rewards balance in the actual NPO situation is negative compared to volunteers, whose rewards from their prosocial behavior usually exceed their flexible contributions to the organizations (Festinger, 1954; Adams, 1963, 1965; Penner et al., 2005).

For that reason, we propose that the following:

Proposition 2: In NPO contexts, paid staff report more conflicts (task, process, status, and relationship conflict) than volunteers and suffer also more negative consequences.

## Materials and Methods

### Participants

We examined a large and representative NPO in Spain, focusing our research on their regional division in the Community of Madrid, composed of 20 local divisions (806 paid staff members and 8,442 volunteers). Although this study focuses on a division, this is a worldwide organization that replies the same functional and hierarchical structure in every country. Therefore, our results can also be applicable to all the divisions of this NPO. Concerning its functional constitution, a strong hierarchical management structure is combined with a democratic decision-making body (the so-called “Committees”) where volunteers occupy the top positions of the organization both at regional and local levels (called “Presidents”). These Committees are responsible for acting in accordance with the general objectives, policy, strategy, and criteria established by the Institution’s higher bodies. However, daily activities and strategic decisions concerning their ongoing projects are made by paid staff. Finally, social projects are run in each local assembly. These projects are developed by teams composed mainly not only by volunteers but also by paid staff. On the one hand, volunteers collaborate with the organization part time with a flexible schedule, depending on their personal circumstances. Also, they have very different profiles in terms of age, professional background, experience working with users, or even seniority in the organization. On the other hand, a reduced number of paid staff are in charge of coordinating these projects, both in terms of administration and social intervention, and also supporting and guiding volunteers on their activity. Most paid staff participants are social workers and psychologists, and have experience dealing with vulnerable collectives and users in social risk.

A total of 60 participants (35 women, 25 men) belonging to different groups of stakeholders in the organization (paid staff and volunteers), working at the headquarters of the NPO in the Comunidad de Madrid and at each local assembly (a total of 10 local assemblies), were part of the collecting data process: 36 paid staff (13 women, 23 men) and 24 volunteers – regular volunteers (8 women, 8 men) and volunteers with management responsibilities, the so-called Presidents (4 women, 4 men). Regarding their educational background, paid staff are university graduates, most of them on health and social sciences. Volunteers report a wide variety of educational background (graduates, professional training, secondary and primary education) and occupations (for example, managers, civil servants, housekeepers, or retired professionals). Regarding participants’ age, on the one hand, paid staff are between 35 and 47 years old and, on the other hand, volunteers are between 19 and 73 years old, which is representative of the diversity in this organization, particularly among volunteers.

### Procedure

Data were collected in two shifts between December 2016 and February 2017 to minimize possible disruptions of the organization’s activities. Qualitative methodology based on focus group was used for several reasons: first, because of the aim of exploring the existent conflicts in non-profit organizational context; second, the heterogeneity of NPO’s activities and projects created doubts concerning the appropriateness of a quantitative analysis; and third, because there was the opportunity of collecting data from the reduced number of volunteers with leaderships responsibilities, which could explain the internal conflict dynamics in depth. A total of seven homogeneous focus-group discussions were organized based on the main groups of stakeholders: four groups of paid staff (from the headquarters and local assemblies), two groups of volunteers, and a group of volunteers with leadership responsibilities (Presidents). Each group was formed by 8–10 key informants who were willing to share their knowledge, experiences, and thoughts regarding conflicts in the organization (Kumar et al., 1993). Participants were invited by e-mail and voluntarily agreed to join the activity that finally took place at the headquarters of the organization. Regarding participant selection, participants were recruited based on the projects ongoing during the data collection (for volunteers and paid staff). Also, paid staff occupying coordinating roles (headquarters) and Presidents (volunteers) were all invited to participate.

During the focus-group discussions, participants described different conflict situations they experienced in their daily work, as well as how they handled them. These discussions were organized following a previously designed semistructured interview, including the following guide questions: (a) presentation of the participants and their role in the organization; (b) organizational structure and work procedures; (c) experience of conflicts in the organization; (d) conflict management strategies; and (e) conflict consequences. Sessions lasted between 60 and 90 min. Confidentiality and anonymity were guaranteed. Afterward, participants gave their consent, and sessions were recorded in audio and transcribed verbatim.

Transcripts were analyzed using the software for coding qualitative data Atlas.ti 7 (Friese, 2013). Following a template analysis approach (King, 2004), a list of codes was defined based on the main themes identified on the focus groups’ transcripts (see Table A1). Some of these codes were defined *a priori*, based on the themes included in the semistructured interview scripts. However, *a posteriori* codes were added while reading and interpreting the texts to complete an exhaustive analysis (King, 2004). To ensure coding reliability, all the authors codified the first transcript separately and then compared their results to standardization purposes. Based on that comparison, codes that differed across these preliminary results were deleted for further analysis, which were carried out by the first author. A total of 93 codes regarding existing conflicts and its consequences were obtained (see Table A1 for the coding list). These codes were grouped in 37 families of codes for reporting purposes.

## Results

Focus-group discussions were highly participative, and results were consistent across the different groups of stakeholders. Most conflicts reported were related to the cooperation between paid staff and volunteers. Among volunteers, those with leadership responsibilities – presidents – reported more conflicts with paid staff than volunteers who collaborate in projects attending users, but also related to role conflict and organizational complexity. Fewer conflicts were reported among paid staff (including hierarchical conflict) or among volunteers. There is an exception for paid staff in a coordinating role (headquarters), which regularly creates communication problems, and who are sometimes considered outsiders by paid staff working at local assemblies due to their focus on administrative issues. Regarding the analysis of the different types of conflicts, the expected four conflicts were reported by paid staff and volunteers: task, process, status, and relationship conflict.

As it was expected in Proposition 1a, task, process, status, and relationship conflicts are identified in non-profit organizational context. Also, as it was proposed in this study (Proposition 1b), relationship conflicts are less reported by both groups of stakeholders. Table 1 presents examples of such conflicts, from both perspectives: paid staff and volunteers. Proposition 2 is also confirmed since, as it was abovementioned, paid staff report more conflicts (task, process, status, and relationship conflicts) than volunteers, identifying also more negative consequences.

**TABLE 1:** Examples of task, process, status, and relationship conflicts described by paid staff and volunteers.

Role in organization	Volunteers	Paid staff
**Conflicts mentioned**		
Task	*“Projects are conditioned by application deadlines, subsidies, budget estimation*… *if this workload is not assumed by a previously established structure, administrative duties surpass taking care of our users.” (S.A., President, Volunteer)*	*“Goals and how we are supposed to achieved them change along the year, then, it is impossible*… *that generates conflict among both paid staff and volunteers. (S.U., Paid Staff)*
Process	*Responsibilities are passing to technical directions (paid staff). And I think that it takes away our prominence, because at the end of the day we forget that this is an entity formed by volunteers (S.A., President, Volunteer)*	*“I think that sometimes conflicts between departments*… *are provoked by the intricate procedures we have to follow*…*” (V.E., Paid staff)*
Status	*”What is government and what is administration? I think that everything is government* (…) *In my opinion, recently, less importance is given to government council (volunteers) work and more importance to paid staff work” (B.E., President, Volunteer)*	*“So many times, the government council (volunteers) made decisions without considering paid staff’s perspective or even asking for our opinion or advice*… *Then, after several months, they conclude the same as we do only in three days working on social intervention.” (A.L., Paid staff)*
Relationship	*“I have close friends in (the organization). However, some people may be your friend, and some people may be not. Those tell me,” You (as volunteer) are here stealing a job position*…*” (S.E., Volunteer)*	*“They’ve got this idea of being a volunteer*… *suddenly they send emails, very annoyed because they are not treated well in the organization, and it was just a misunderstanding*… *(B.L., Paid staff)*

As Table 1 shows, both paid staff and volunteers report all types of conflict; however, these are not precisely about the same issues. It is also important to note though that, depending on the hierarchical and functional position, participants’ reasoning about the conflict issues differs. That is the case for volunteers and paid staff, whose perspectives are occasionally even opposite.

### Task Conflict

#### Project Coordination Complexity, Task Diversity, Deadlines, Task Prioritization, and Quality of Attention

##### Paid staff

*“This is a complex organization, we are lot of people, we manage a lot of things and, above all, everyone looks for immediacy” (R.A., Paid Staff, headquarters)*. Participants highlight the NPO’s complexity, in terms of internal structure, different collectives working together, and task diversity. Besides their work as social workers or psychologists, paid staff are responsible for administrative tasks (e.g., project justification) to obtain the financial support. This is a very demanding and time-consuming task; procedures are constantly changing, and they feel overwhelmed by deadlines. They are requested to register quantitative information using changeable and complex software programs, which even duplicate processes: *“We live under pressure: this data must be in the Economic Department right now. And I have 50 users here that I must attend” (M.U., Paid Staff).*

In this regard, paid staff and volunteers dissent from the headquarters’ management regarding the prioritization of issues. From the headquarters, project justification is highlighted as a priority to maintain the financial support needed to carry out their social labor; however, paid staff, in the same line as volunteers, would like to focus on social intervention, preventing a decrease on the quality of the attention they provide to users.

##### Volunteers

These conflicts, specially working toward a deadline and the project’s coordination, are reported also by volunteers, particularly by Presidents, who also deal with administrative and coordination duties. They are aware of task diversity and the importance given by the regional office to project justification, but they are also concerned about the quality of users’ attention sharing the same perception as local paid staff; attending users should be their priority.

“The problem is that there is a lot of work that is being devoted by social workers to fulfill administrative tasks instead of being focused on social intervention” (S.A., President, Volunteer).

#### Lack of Personnel and Resources

##### Paid staff

*“If these are the resources, we have to work in a different way to achieve everything that is asked from us, we have to increase our own resources” (A.R., Paid Staff)*. Paid staff pointed out that they must be in charge of several activities during their work day due to the organization’s lack of personnel and economic resources, *”if we plan an activity, and we are supposed to be seven, but then we are three, or less, and someone gets sick… that is our daily routine” (I.M., Paid Staff).*

They are also worried about how would volunteers cope with difficult and emotional situations with users; due to their lack of training or previous experience, it is more likely that they made wrong decisions allocating resources among users:

”A volunteer, who is the sixth time that he or she faces that situation, obviously stands up for users because he or she feels closer to them than us… delivering an emergency aid and then the organization gives forty euros that was meant to be for food” (E.L., Paid staff).

##### Volunteers

“*You can have 21 paid staff employees in a project team today, none in 3 months and then 20 different employees” (S.A., President, volunteer)*. Presidents described how they usually invested time and effort creating well-coordinated teams as well as training paid staff in specific issues of the project. However, due to the lack of personnel also at the regional level of the organization, well-trained paid staff employees are transferred to the regional assembly. Therefore, local teams must start again bringing together a new team:

“We have invested a lot of work creating teams, and, when that employee is already trained, he or she is transferred to a different local or regional assembly” (A.E., President, Volunteer).

#### Lack of Communication and Commitment

##### Paid staff

“I’ve lived many situations, and nothing has been finally achieved… there is a lack of commitment, maybe from both sides, but above all, from management itself” (I.M., Paid Staff).

Due to communication deficiencies and the perception of inactivity of the regional assembly, distrust is a main source of conflict. Paid staff and volunteers distrust regional assembly’s management capacity and do not agree with the communication policies, not only regarding internal issues but also about how information is made public (e.g., malicious rumors about their charity activities not refuted).

Overall, the head office is not consistently implementing new procedures or activities to promote well-being and job satisfaction among paid staff and volunteers, and it is usually unapproachable to transfer problems’ information. This situation increases distrust and emotional discomfort among the members of the organization as well as an overall lack of credibility of regional management. The head office is aware of this problem, however, managers there blame the organization’s multitasking method: paid staff are working on different projects simultaneously, receiving instructions and requirements from different supervisors who set their own priorities, and there is no communication between them.

##### Volunteers

Since volunteers especially identify themselves with the organization’s goals and values, they feel personally attacked when it is criticized, or it suffers any discredit accusation not being refuted by the headquarters. *“It is essential that higher hierarchical levels transmit the values of this organization, especially when its public image is attacked” (F.E., Volunteer).*

Volunteers also consider that additional conflicts (such as paid staff transferring among assemblies) influence paid staff commitment, both with their tasks and with the organization. Therefore, sometimes it is extremely hard to find someone that may help them out when there is a problem, or they need advice.

”If managers are constantly changing, we all wait for someone else to take charge on things, so they never get done… Here nobody takes the phone. There is a great lack of communication” (S.E., Volunteer).

### Process Conflict

#### Undefined Tasks

##### Paid staff

“*Undefined tasks generate daily conflicts among teams” (J.E. Paid Staff, headquarters)*

Paid staff must adopt a multitasking approach to cope with the highly diverse task flow. Furthermore, both volunteers and paid staff report that the lack of personnel stands in the way of efficiency. Thus, volunteers are required to support paid staff to offer an appropriate attention to users and meet financial justification requirements, “*many times we asked volunteers to help us to finish administrative duties instead of what they would like to do, that is helping users” (C.R., Paid Staff).*

##### Volunteers

Volunteers, on the contrary, consider that their role should be mainly to attend users, while paid staff, who receive economic retribution and are responsible for the NPO’s activity, should also accomplish administrative tasks.

“Volunteering moves the organization. I have noticed in a way, the idea that volunteers work or have to do paperwork, making paid staff’s lives easier and it should be just the opposite” (Y.O., Volunteer).

#### Role Conflict and Role Ambiguity

##### Paid Staff

“There is A Problem, Which is Caused By… Economic Crisis. Less Money Means Less Paid Staff, So We Recruit Volunteers” (E.L., Paid Staff). Due to the recent economic crisis, NPOs’ hiring capacity decreased dramatically. Therefore, volunteers’ activity has supported the organization more than never.

Paid staff consider volunteers’ contributions essential, however, they are also worried about their job positions; considering the voluntary character of the organization, their perceived role ambiguity, and the lack of economic resources, they are concerned about being replaced by “unpaid” volunteers. Furthermore, they are concerned about how volunteers may cope with users’ problems, without specific training, since it is a high emotional and demanding work that should be managed carefully.

###### Volunteers

”*There are people who do not want volunteers; because in the end we let ourselves be handled by users… We distort their work a lot, they do not see us as a help” (P.A., Volunteer).*

Although they understand paid staff’s reservations, overall, volunteers consider that they are very capable of attending users. Indeed, they demand more responsibilities on this matter, since volunteering is one of the main pillars of the institution’s ideology. Moreover, volunteers argue that users may perceived that paid staff members are acting as professionals instead of just providing social support, so they can establish a closer relationship with them. “*As volunteers, we want our role to be more interactive with users because I think that they may be more comfortable talking to us, than with someone with more authority” (A.R., Volunteer)*.

Although volunteers’ and paid staff’s opinions differed regarding their role with users, both demand specific training. Each project has specific characteristics that require different competences, which are usually not covered during their initial training.

### Status Conflicts

#### Hierarchical Structure and Leadership Based on Power

##### Paid staff

“*Ours is a top-heavy hierarchical structure” (O.L., Paid Staff)*. The very structured and vertical hierarchy hinders both decision-making processes and communication. Paid staff explain that leadership is usually based on power, and decisions made at higher hierarchical levels must be obeyed even when that entails changes in the normal functioning of the organization: “*Instructions are passed by from the higher levels of the hierarchical structure and it is not egalitarian under any circumstances… the basis of this type of leadership is fear” (O.L., Paid Staff).*

##### Volunteers

Overall, local volunteers do not perceive that the prevailing leadership based in the organization is based on power. However, presidents report conflicts related to the very hierarchical structure of the organization that prevent top levels of knowing the local assemblies’ reality.

“Those at the top do not share the same vision. They must be aware that the national, regional and local structure are different, because unlike them that usually work with paid staff, I’m working mostly with volunteers” (C.M., Volunteer, President).

#### Distrust on Head Office Management

##### Paid staff

Paid staff consider that they can provide first-hand information about intervention and users’ needs, so they should contribute to decision-making processes in a more active way. In fact, not being able to do so is very stressful and even frustrating: *“When ‘higher’ levels plan everything, no one asks us, so we have the feeling that we are just services’ vending machines” (O.L., Paid staff).*

Overall, strategic decisions are made by head office’s personnel, who are usually not aware of each local assembly’s particularities. Due to the lack of communication between hierarchical levels, relevant information to plan interventions and distribute resources is missing. Thus, when there is a problem inside the organization or even situations that need to be promptly sorted, it is not easy to find out who should make the decisions to solve it.

Nevertheless, regional staff managers consider that sometimes, participation in decision-making processes is taken for granted. It is seen as a right instead of as a deference or positive characteristic whose aim is to create a democratic work environment where everybody can contribute: *“In this organization we have made participation and consensus something that instead of being a positive attribute, ends up becoming a requirement: if I do not participate and I make decisions I do not get involved” (B.L., Paid staff, headquarters).*

##### Volunteers

Volunteers who are part of the government body (Presidents) claim that, according to their role expectations in a volunteering organization, they should be able to participate in strategic decisions in their local assemblies. Instead, they receive instructions from regional paid staff, who only gather information about social intervention from paid staff or during periodical meetings:

“As local president, I make non-important decisions on a daily basis, because the guidelines, relevant decisions, rules, and objectives are determined by the Head Office (run by paid staff)” (I.D., President, Volunteer).

#### Performance Evaluation, Poor Feedback, and Ways to Transmit Problems

##### Paid staff

Paid staff consider that the organization is reorienting their performance evaluation, basing it on quantitative parameters instead of qualitative ones: *“I think that sometimes our supervisors’ value more how do we organize administrative tasks instead of what really is our aim here and why we studied our degree, social intervention, and working with users” (M.O, Paid staff).*

Additionally, they point out that they do not receive enough feedback during the evaluation process, and they are not able to give feedback to their supervisors. Although the evaluation procedure was supposed to be a 360° evaluation, it is vertical and top–down. Therefore, problems are not solved since the responsible person is not even aware of them.

“It is a pity, because the idea of being evaluated… is interesting… a way of improvement…, but if there is a two-way flow communication” (E.M., Paid staff).

##### Volunteers

“*We are not evaluated, they just say you are it doing wrong” (A.L., Volunteer, President*). Both paid staff and presidents (volunteers) explain that not only it is difficult to transmit their concerns to higher hierarchical levels, but also they claim for their constructive feedback. Based on the performance evaluation procedures, they consider that only their mistakes are highlighted without having the chance to discuss which problems or difficulties they have.

#### Power Imbalance

##### Paid staff

Paid staff explain that volunteers are more powerful since they are protected by the organizational vision (voluntary organization), values, and even policies. Thus, paid staff perceive their contract as a bond that entails not only obligations but also detrimental consequences in terms of power imbalanced compared to volunteers; they can leave the organization whenever they want or issue a complaint against paid staff without assuming any occupational risk. Overall, they considered that, when there is a conflict situation, volunteers’ rights may act against paid staff’s rights:

“When a volunteer confronts you or questions your work, many times you do not have capacity to confront him. Because they have nothing to lose” (P.A., Paid Staff).

##### Volunteer

On the contrary, volunteers consider that, because they are employees, paid staff must cope with certain tasks, but they are also supported by HR and they can demand changes by means of their labor contract. In return, they consider that paid staff are responsible for keeping the organization functioning smoothly:

”They are the ones who have the power, that are remunerated, and they always know what their job is… each of us have our status. I am here to offer my time, and my help. If the situation does not convince me, I leave… In return, you can call HR department saying I want to be in a different project, I do not feel good where I am” (P.A., Volunteer).

#### Paid Staff Transferring

##### Paid staff

*“As an organization, we have not got a clear procedure to follow in certain conflicts; therefore, it is solved by transferring the people involved to a different project team” (I.S., Paid Staff)*. Paid staff transferring is not only used as a strategy to relieve the lack of personnel; paid staff consider it as also used as an avoiding conflict management strategy. The organization transfers those members involved in team conflicts to another assembly, instead of trying to find a solution that could prevent its reoccurrence. Consequently, on occasion, those teams receiving staff transfers decrease their performance and motivation, since new members do not fit with the group or they create new problems, so conflicts spread out to different teams*: “There are people who work great, who are super motivated, and you can see that, because of a transfer, that person is decreasing his or her motivation, and passing on that demotivation to the team” (J.E., Paid staff).*

Paid staff reported an additional problem related to personnel transferring; when they truly want to leave a certain team, they do not receive any answer to their petition from management. Therefore, they feel that the organization does not look after their needs and does not listen to their requests; *”there are workers who are caught up in their positions, we are not transfer anywhere else even if we ask for it (I.S., Paid staff).*

Although this problem is also detected at the regional level, conflict escalation seems to increase their difficulties to find a straightforward solution and they solve it by moving those employees involved:

”Sometimes if it would had been detected before or if it would had been discussed, I think it could have been solved, could it? Now there is a situation of tension, of labor conflict, because people had to be transferred, because now they are afraid” (R.A., Paid Staff, headquarters).

##### Volunteers

Volunteers perceived that these transfers affect paid staff’s commitment with the organization and the task they oversee. Although the number of volunteers may buffer the negative effect of their changeable situation, that is not the case of paid staff who are employed by the organization and should fulfill certain duties. Volunteers explain that their personal and professional circumstances affect how much time they can spend collaborating with the NPO; *“our strength is that there is a lot of volunteering, and our weakness is that, finally, volunteers, no matter how much committed they are, have obligations outside the organization, with its ups and downs” (Y.O., Volunteer).*

### Relationship Conflict

#### Personal Disagreements and Unresolved Personal Issues

##### Paid staff

“We are like a big family; we do not always get along with each other and we usually do not agree with the inheritance” (M.T., Paid staff). Paid staff explained that volunteers’ personal characteristics usually lead to conflict in their teams. Since there are no selection procedures in the organization to either accept or assign volunteers to projects, only their personal preferences, sometimes they do not fit with the team or they try to impose their own way of doing things. Under these circumstances, paid staff explain that, due to their volunteer status, they manage these misunderstandings reporting their complaints to the highest hierarchical levels, denouncing that they are not feeling well treated by paid staff. This behavior is considered by paid staff as a lack of respect and recognition of their work, even generating resentment among team members.

“Volunteers have their own ideas and some of them try to impose them…there are troublesome volunteers, very difficult ones, or those whose their personal characteristics just do not fit in the project or local assembly and if we do not change our way of doing our work based on their ideas they create problems in the team” (A.R., Paid staff).

##### Volunteers

*“Do not tell me what I have to do, if you are burnt out do not pass your frustration on me” (S.E., Volunteers).* Volunteers explain that paid staff sometimes see them as a threat, someone that can replace them for free supported by the organization’s values, as it was previously mentioned. These circumstances not only lead to role conflict and role ambiguity but also create personal incompatibilities between them.

“I have close friends in (the organization). However, some people may be your friend, and some people may be not. Those tell me, ‘You (as volunteer) are here stealing a job position…”’ (S.E., Volunteer).

### Consequences of Conflicts

#### Frustration and Anxiety

##### Paid staff

“*Some people are so frustrated” (I.S., Paid staff)*. Paid staff report a frustration feeling stemmed from work overload, for example, due to the slow pace of administrative duties and the lack of trust in regional decision-making processes. They are not able to transmit their concerns or doubts to higher hierarchical levels due to the lack of direct communication channels; therefore, they are very insecure if they have to act without consulting with their supervisors what should be done, which occurs quite often due to the urgency of their activity with users: *“I think that HR department usually do not know what their own employees do and the difficult situations they have to deal with” (I.S., Paid staff).*

##### Volunteers

Volunteers explain that, in some way, paid staff transfer their anxiety and stress to volunteers in an unconscious manner. Since they feel pressured by their working conditions and the obligatory nature of their tasks, they discharge their frustration on volunteers, increasing their demands instead of supporting them:

”I understand that a person has to channel those problems or manage them somehow, but it overloads and stress out volunteers, that is the reason why some people decide to leave this organization and go to a different entity” (Y.O., Volunteer).

#### Insecurity to Act Without Consulting, Fear of Reprisals, Lack of Motivation, and Emotional Exhaustion

##### Paid staff

Besides suffering long-term frustration and anxiety, paid staff feel vulnerable and not supported or protected by the organization, even afraid of suffering reprisals if they make a mistake: *“our stress level is quite high since we are constantly trying to transmit our problems and concerns but that they have not listened to us for a long time” (O.L., Paid staff).*

Therefore, teams’ motivation, organizational commitment, and satisfaction with their activity decrease, and overall team emotional exhaustion takes place: *“if they do not watch over you, how are you going to look after those you are supposed to be taking care of?”(I.S., Paid staff).*

*“Volunteers are lost, paid staff have a different nuance” (E.V., Paid staff)*. Due to their different position in the organization, paid staff’s and volunteers’ responses to this lack of job satisfaction are quite different. Paid staff generally try to cope with the situation during extended periods of time in order to keep their job position, which leads to a transferring request or even absenteeism.

“Losing volunteers and losing workers, because workers do not get lost because they leave the organization. In my previous position, I lost motivation and, therefore, I was less useful…they lost me as a worker” (D.A., Paid staff).

##### Volunteers

When volunteers are not satisfied with their activity in the organization, they do not perceive an obligation to stay there under unpleasant circumstances. Due to their commitment values and motivation to help, they try to carry on asking also for a new project to collaborate with, but if their situation does not change, they just leave the organization.

“I am here to offer my time and my help, if the situation doesn’t convince me, I leave” (P.A., Volunteer).

**FIGURE 1:**
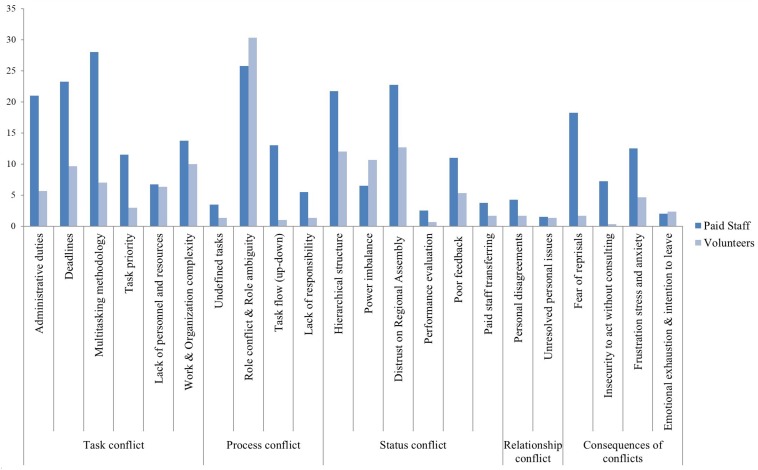
Incidence of most reported conflicts and their consequences (based on the number of cites) by paid staff and volunteers.

Additionally, as stated in Proposition 2, we also analyzed who report more conflict and who will suffer more negative consequences derived from this experience of conflict. In this regard, as Figure 1 shows, results support this proposition; overall, paid staff experience more conflicts (considering the fourth types analyzed) and, as expected, report suffering more negative consequences due to this conflict experience. In NPOs’ contexts, paid staff report more conflicts (task, process, status, and relationship conflicts) than volunteers, suffering also more negative consequences.

Power imbalance together with role conflict, role ambiguity, and intention to leave are the most reported conflict issues among volunteers. However, paid staff are more concerned about administrative duties and multitasking methodology, although they also report role conflict and role ambiguity as a main cause of conflict, since they would expect their job to be based on their academic and professional background instead of being related to administrative tasks. Compared to volunteers, paid staff reported higher levels of fear of reprisals and insecurity to act without consulting.

Among paid staff, those working with volunteers as part of same teams at local assemblies are the groups reporting more conflicts, particularly related to role conflict. Among volunteers, presidents, due to their managerial role, report more conflicts that local volunteers. Indeed, presidents report similar levels of role conflict and distrust on the headquarters to paid staff, illustrating the effects of the intricate power dynamics and hierarchical complexity in the organization.

Based on these results, our two propositions are supported. Paid staff and volunteers differed on their perceptions of these types of conflicts, their prevalence, and causes: paid staff not only perceive more conflicts but also report more negative consequences. Table 2 shows a summary of the main specific conflict issues and consequences reported by our participants.

**TABLE 2:** Conflicts detected in NPOs considering traditional conflict taxonomies and their reported negative consequences.

**Types of conflicts**				**Negative consequences of conflicts**
	
**Task conflict**	**Process conflict**	**Status conflict**	**Relationship conflict**	Frustration/anxiety Lack of motivation Insecurity to act without consulting Fear of reprisals Absenteeism Emotional exhaustion/intention to leave

Deadlines Quality of attention Administrative duties Task prioritization Multitasking methodology Project coordination’s complexity Lack of personnel/resources Commitment Work complexity Lack of communication	Assumption of responsibilities Actual and expected functions of paid staff and volunteers (Role conflict) Role ambiguity Undefined tasks Task flow (up-down) Lack of responsibility Differences between assemblies	Paid staff transferring Evaluation differences Performance evaluation Poor feedback/ways to transmit problems Power imbalance Regional assembly’s inactivity Distrust on regional assembly’s decision-making processes Regional assembly’s competences and knowledge about local assemblies Leadership based on power Hierarchical structure Local decisions made at regional level	Personal disagreements Unresolved personal issues: – Lack of respect – Lack of recognition – Resentment	

## Discussion

The main objective of this study was to progress on the previous research findings analyzing conflicts in NPOs, considering as theoretical framework several consolidated organizational psychology theories: on the one hand, the conflict theory (Deutsch, 1973) to analyze how conflicts arise in this context, and, on the other hand, the social comparison theory (Festinger, 1954) and the equity theory (Adams, 1963, 1965) to analyze the differences between paid staff’s and volunteers’ perceptions regarding these conflicts, as well as their consequences.

This study demonstrates that, in order to understand NPO conflicts, we need an extended conflict taxonomy: task, relationship, process, and status conflict. The well-known taxonomy of organizational conflicts that differentiate between task, relationship, and process conflict (Jehn, 1995) is insufficient to understand the conflict nature in NPOs. Our results also highlight the importance of how these conflicts develop in NPOs and which situations or issues trigger their appearance, providing the opportunity to not only manage but also prevent conflict situations in this organizational context by means of understanding the phenomenon and how it is developed. This is particularly relevant considering NPO particularities such as work organization, role dynamics, and contributions–rewards balance between paid staff and volunteers, which make the difference and create the perfect “breeding ground” to conflicts.

For NPOs, maintaining and protecting their reputation and donors’ trust is essential in order to guarantee, to a greater extent, their projects’ survival (Müller-Stewens et al., 2019); therefore, internal conflicts are concealed. However, there are two main factors that are increasing research interest in this issue and therefore evincing the problem. First, volunteers are essential to NPOs’ labor; otherwise, they would not be able to reach their organizational goals only by means of employees (Englert and Helmig, 2018) and, by extension, governments that rely on these organizations to attend social issues (Henriksen et al., 2012). The second factor is the professionalization of NPOs and the implementation of managerial practices to guarantee their efficiency, which usually clashes with their volunteering values (Ganesh and McAllum, 2012). In this regard, our results analyzing these two stakeholders’ perspective are indeed consistent with their previous studies analyzing paid staff’s and volunteers’ interaction as a trigger of conflict in NPOs (Macduff, 2012; Rimes et al., 2017). However, our study goes a step further identifying and labeling the existing types of conflicts provoked by these dynamics and these organizations’ characteristics and, therefore, facilitating that actions to prevent or manage them are promoted in NPOs.

In this regard, work overload and work dynamics, including resources investments, are promoting both task and process conflicts. The implementation of business-like policies (Maier et al., 2016) as part of a “professionalism” or “managerialism” trend in NPOs (Kreutzer and Jäger, 2011; McAllum, 2018) is increasing not only task conflicts due to the implementation of multitasking methods but also administrative duties and deadlines. The lack of personnel and resources as well as communication deficiencies make this situation worse. Process conflicts are mainly provoked by both paid staff’s and volunteers’ role ambiguity and role conflict who are not sure of their exact role in the organization and even feel they are fulfilling the duties of two different positions. Work dynamics and shortage of resources together with decision-making processes lead to status conflicts in NPOs. The very hierarchical structure in this organization prevents feedback between different levels and promotes power imbalance perceptions, uncertainty, and distrust not only between paid staff and volunteers but also between different hierarchical levels. However, although it is reported by both stakeholders, among these four types of conflicts, relationship conflict is the least reported conflict by both volunteers and paid staff. This result is remarkable since it highlights that, although our results also coincide with previous studies regarding volunteers and paid staff identity differences (Kreutzer and Jäger, 2011) that may create personal incompatibilities, in NPOs, the importance of values and engagement with the social aim of the organization can be considered a protective factor against conflicts’ negative consequences, especially for volunteers. An interesting future line of research is the analysis of how the type of job protects employees to the appearance of relationship conflicts. Data in this study suggest that working on social issues with vulnerable people (refugees, children, human trafficking, etc.) protects employees from negative relationship conflict since they give priority to their common goals instead of to their personal incompatibilities. Therefore, reflecting on the equity theory (Adams, 1963, 1965), for relationship conflict, the contributions–rewards balance is closer to equilibrium than for those conflicts related to work processes and power position in the organization. This effect is also demonstrated by the fact that both groups report low levels of turnover intention, despite the high rates of conflict reported, especially for paid staff.

Likewise, regarding the identities’ differences and the social comparison between volunteers and paid staff, both stakeholders demand, to a certain extent, leading the organization: volunteers based on organizational values and position, and paid staff based on their expertise (Munduate and Medina, 2017). This also relates to motivation and expectations; when managerial structures are introduced in this kind of organization, particularly volunteers report that administrative duties are not their aim or even responsibility, as a natural reaction to maintaining their great positive balance between their contributions and the emotional rewards obtained from volunteering.

Overall, results show that paid staff report more conflicts than volunteers. Indeed, based on the number of reported conflicts, it can be concluded that paid staff are absorbed in a loss conflict spiral, starting with workload and administrative duties, getting worse with discrepancies regarding decision-making process and ending with fear of reappraisal and anxiety, even considering their employment contract as a disadvantage, which forces them to stay in the organization. Paid staff report the greatest number of conflicts for all types of conflicts, but especially for multitasking methodology and administrative duties and role conflict. These results highlight the importance of considering paid staff perspective when analyzing conflicts in NPOs.

Paid staff indeed experience more conflicts than volunteers. Even so, previous research has traditionally put a spotlight on volunteers, overshadowing both their needs in their working context and the consequences of leaving them out. Volunteers, on the contrary, who are free to leave the organization, decide to stay because their social contribution is more important for them than those conflict situations they may experience in the organization. An exception in this group are Presidents, volunteers with managerial responsibilities, who identify more conflicts than local volunteers and even similar or more conflicts than paid staff, on certain issues. This difference can be explained by the nature of their job that implies fundamentally different power positions toward paid staff; Presidents consider that paid staff with management duties at the headquarters are the ones who really plan all the lines of action without involving them on their decisions, and simultaneously, they deal with volunteers’ coordination.

These results also reflect the effects of the intricate role dynamics that prevails in many NPOs, and the consequences of professionalization, not only for volunteers, who report higher levels of role ambiguity (process conflict) due to this transition, but also paid staff, since they have to deal with the administrative processes. In this regard, considering the organization’s voluntary character, Presidents, who are volunteers with management responsibilities, feel that their managing role is being taken away by paid staff. Moreover, paid staff do not agree on focusing on administrative tasks instead of attending users, since they consider it as not congruent with their formal training.

Therefore, regarding the consequences of conflict, findings suggest that paid staff suffer more negative consequences than volunteers, mainly frustration, stress, and anxiety, whereas emotional exhaustion and turnover intentions are low. As it was abovementioned, it is interesting to remark the low level and intensity of the relational conflicts perceived both by the paid staff and the volunteers. The negative consequences that this type of conflict has for the organizations and those who suffer from it are well known (Medina et al., 2005). Thus, this may explain the low levels of emotional exhaustion and turnover intention for both groups, paid staff and volunteers (Benitez et al., 2011). It is also relevant how this NPO’s prosocial work seems to protect workers from relationship conflict and their negative consequences, such as the desire to leave the organization or emotional exhaustion.

### Practical Implications

Our results suggest several practical implications. First, organizations where paid staff and volunteers work together, such NPOs, should be aware of the existence of task, process, relationship, and status conflicts in their context and particularly between these two main stakeholders. Second, these conflicts are embedded in the organization’s procedures and characteristics; the structure, work nature, and even the understanding of organizational values promote them. Therefore, using our results as a checklist, supervisors and team leaders can identify and therefore manage adequately or even prevent these conflicts. Third, it is important for HR departments to focus in the situation of paid staff, who deserve special attention. Although the focus has been traditionally put on volunteers, based on our results, paid staff perceive more conflicts and have a more negative situation in organizations. Thus, as a general advice, paid staff in NPOs should be paid not only a salary but also attention and care from their organization.

### Potential Limitations and Future Studies

This study has some limitations that must be considered in order to generalize the obtained results. This study was conducted in an only NPO branch, so future studies should replicate the analysis in different NPOs to confirm that our conclusions are applicable to different organizations. Nevertheless, as a positive aspect of our participant NPO, this is a worldwide organization that replies the same functional and hierarchical structure in every country, so these results can potentially be generalized to all these divisions around the world. Moreover, despite the possible differences among NPOs, our results are consistent with previous studies. Furthermore, qualitative data limit the explanatory potential of the study and the number of participants involved in the research. However, they also provided the opportunity to understand and explore the complexity of NPOs and the existing conflict dynamics in this singular work environment. Future studies should analyze conflicts in these organizations using also quantitative approaches that make possible to conduct a deeper analysis of conflict situations and those variables influencing them.

## Conclusion

This study constitutes a remarkable contribution to NPOs’ knowledge from a theoretical and practical perspective. From a theoretical view, this study not only analyzes specific types of conflict in NPOs (task, process, status, and relationship conflict) but also considers doing so from paid staff’s and volunteers’ different perspectives. Additionally, it is built up on three consolidated theories: the conflict theory (Deutsch, 1973), the social comparison theory (Festinger, 1954), and the equity theory (Adams, 1963, 1965). From a practical perspective, some general recommendations to NPOs can be extracted from our results. First, explicitly identifying conflicts in these organizations helps parties to manage them; however, training is crucial, particularly for those who have management responsibilities, usually paid staff. NPOs’ leaders can prevent or minimize these conflicts and their consequences by introducing some routines in the organization, such as monitoring paid staff’s and volunteers’ relationship, combining qualitative and quantitative data. Second, it must be taken into consideration the organizational changes that NPOs are facing (e.g., decrease in economic resources, professionalization), which results on paid staff not only experiencing more conflicts but also suffering more negative consequences. These organizations should especially be concerned about paid staff’s needs, as much as for volunteers’, in order to guarantee the achievement of their social aims. Overall, promoting transparency between stakeholders and training to overcome differences would contribute to improving NPOs’ functioning both internally and externally.

## Data Availability Statement

The datasets for this article are not publicly available due to funding requirements until the end of the ongoing research project. Requests to access the datasets should be directed to the corresponding author.

## Ethics Statement

The studies involving human participants were reviewed and approved by the Ethics Committee of the Faculty of Psychology (University of Seville, Spain) and the Ethics Committee of the participant non-profit organization. Written informed consent was not provided because participants agreed to join the study upon invitation after receiving all the information regarding the research purpose, procedure and data treatment. They gave their explicit informed consent during the Focus Groups sessions recordings.

## Author Contributions

All the authors contributed to the conception and design of the work and the acquisition, analysis, and interpretation of data. They drafted the work and revised it critically. The authors gave the final approval of the manuscript before the submission. They participated at every stage of the research process.

## Conflict of Interest

The authors declare that the research was conducted in the absence of any commercial or financial relationships that could be construed as a potential conflict of interest.

## Appendix

**TABLE A1:** List of *a priori* and *a posteriori* codes.

***A priori* codes**	***A posteriori* codes**	
Conflict regarding decision-making processes Relationship conflict in teams Conflicts between volunteers and paid staff ***Empathy between working positions** Volunteers and paid staff roles Volunteers’ power Work complexity (working with users) Conflict escalation Organizational complexity Project’s coordination complexity Deadlines Differences between local assemblies Communication Training Leadership Planning Volunteers temporariness Decision-making processes Organizational members’ vocation Time management Differences in conflicts due to seniority Quality of attention	Take others’ responsibilities Commitment Distrust on headquarters’ decisions Lack of headquarters’ competences **Lack of empathy between working positions** Insecurity to act without consulting Discomfort among team members Participation and consensus as an obligation Privileges as rights Lack of responsibility Undefined tasks Volunteers’ role enrichment Absenteeism Volunteers’ attitude Positive aspects of the evaluation Organizational change Positive consequences of change of management Negative consequences of volunteers’ inexperience Inactivity and lack of awareness of regional assembly Paid staff vulnerability Evaluation differences Difficulties in the organization and coordination of events Burned out teams Stress and anxiety Vertical evaluation of performance Lack of alternatives to transmit problems Lack of coordination Lack of effectiveness of management Lack of spaces Lack of feedback between hierarchical levels Lack of intervention methodology Lack of motivation Lack of paid staff Lack of resources	Lack of institutional socialization Lack of volunteers Communication Promotion of labor climate Volunteer training Paid staff training Frustration Schedules that differ from the established timetables Public image Paid staff’s involvement Volunteers’ emotional labor Relationship with management Importance of paid staff Importance of volunteering Influence of personal characteristics Improvement detected Project justification President’s role Slowness, lack of reaction of the organization Amount of information managed Organizational improvements Multitasking methodology Need of enrichment of meetings Transferring responsibilities Prioritization of the activity Different local realities for training Cut back on social intervention Resistance to change Paid staff supporting volunteers Rumors Volunteer satisfaction Somatization Duplicate tasks Administrative duties Paid staff transferring Task flow (up-down) Quantitative assessment Volunteers acting as paid staff

*(*) This a priori code was modified and replaced by the a posteriori code in bold font.*

## References

[B1] Adams J. S. (1963). Toward an understanding of inequity. *J. Abnorm. Soc. Psychol.* 67 422–436. 10.1037/h0040968 14081885

[B2] Adams J. S. (1965). “Inequality in social exchange,” in *Advances in Experimental Social Psychology*, ed. Berkowitz L., (New York: Academic Press), 267–299.

[B3] Alcock P. Kendall J. (2011). Constituting the third sector: processes of decontestation and contention under the UK Labour Governments in England. *Voluntas Int. J. Volunt. Nonprof. Organ.* 3 450–469. 10.1007/s11266-010-9178-9

[B4] Alfes K. Shantz A. Saksida T. (2015). Committed to whom? Unraveling how relational job design influences volunteers’ turnover intentions and time spent volunteering. *Voluntas* 26 2479–2499. 10.1007/s11266-014-9526-2

[B5] Ariza-Montes A. Giorgi G. Leal-Rodríguez A. Ramírez-Sobrino J. (2017). Authenticity and subjective wellbeing within the context of a religious organization. *Front. Psychol.* 8:1228. 10.3389/fpsyg.2017.01228 28769854 PMC5516145

[B6] Ariza-Montes A. Roldán-Salgueiro J. Leal-Rodríguez A. (2015). Employee and volunteer: an unlikely cocktail? *Nonprof. Manag. Leadersh.* 25 255–268. 10.1002/nml.21121

[B7] Ariza-Montes A. Tirado-Valencia P. Fernández-Rodríguez V. Hager M. A. (2018). Religious vs secular volunteering motivations: a study on European elders. *Res. Ageing Soc. Policy* 6 82–111. 10.4471/rasp.2018.3136

[B8] Baluch A. M. (2012). *Human Resource Management in Nonprofit Organizations. Routledge Studies in the Management of Voluntary and Non-Profit Organizations.* New York, NY: Routledge.

[B9] Bendersky C. Hays N. A. (2012). Status conflict in groups. *Organ. Sci.* 23 323–340. 10.1287/orsc.1110.0734

[B10] Benitez M. Medina F. Munduate L. (2018). Buffering relationship conflict consequences in teams working in real organizations. *Int. J. Confl. Manag.* 29 279–297. 10.1108/IJCMA-11-2017-0131

[B11] Benitez M. Medina F. J. Munduate L. (2011). Studying conflict in work teams. A review of the Spanish scientific contribution. *Papeles Psicól.* 32 69–81.

[B12] Berzin S. C. Camarena H. (2018). *Innovation from Within: Redefining How Nonprofits Solve Problems.* New York, NY: Oxford University Press.

[B13] Blake J. (2012). “Professionalism and the third sector,” in *Proceedings of the Communication at the Annual Meeting of the ISTR 10th International Conference*, Bundoora.

[B14] Brandsen T. Van de Donk W. Putters K. (2005). Griffins or chameleons? Hybridity as a permanent and inevitable characteristic of the third sector. *Int. J. Public Admin.* 28 749–765. 10.1081/pad-200067320

[B15] Brown W. A. Yoshioka C. F. (2003). Mission attachment and satisfaction as factors in employee retention. *Nonprofit Manag. Leadersh.* 14 5–18. 10.1002/nml.18

[B16] Cabra de Luna M. A. (2016). Realidad del Tercer Sector en España y crisis del Estado de Bienestar: retos y tendencias. *Revi. Int. Polít. Bienestar Trabajo Soc.* 1 115–134. 10.15257/ehquidad.2014.0005

[B17] Chenhall R. H. Hall M. Smith D. (2016). Managing identity conflicts in organizations: a case study of one welfare nonprofit organization. *Nonprofit Volunt. Sect. Q.* 45 669–687. 10.1177/0899764015597785

[B18] CIRIEC (2012). *The Social Economy in the European Union.* Brussels: European Social and Economic Committee.

[B19] Cnaan R. A. Handy F. Wadsworth M. (1996). Defining who is a volunteer: conceptual and empirical considerations. *Nonprofit Volunt. Sect. Q.* 25 364–383. 10.1177/0899764096253006

[B20] Corral-Lage J. Maguregui-Urionabarrenechea L. Elechiguerra-Arrizabalaga C. (2019). An empirical investigation of the Third Sector in Spain: towards a unified reconceptualization. *Rev. Contabil.* 22 145–155.

[B21] Corry O. (2010). “Defining and theorizing the third sector,” in *Third Sector Research*, ed. Taylor R., (London: Springer), 11–20. 10.1007/978-1-4419-5707-8_2

[B22] De Wit F. C. Greer L. L. Jehn K. A. (2012). The paradox of intragroup conflict: a meta-analysis. *J. Appl. Psychol.* 97 360–390. 10.1037/a0024844 21842974

[B23] Deutsch M. (1973). *The Resolution of Conflict: Constructive and Destructive Processes.* New Haven, CT: Yale University Press.

[B24] DiMaggio P. J. Anheier H. K. (1990). The sociology of nonprofit organizations and sectors. *Annu. Rev. Sociol.* 16 137–159. 10.1146/annurev.so.16.080190.001033

[B25] Eisenberg E. M. Eschenfelder B. (2009). “Applied communication in non-profit organizations,” in *Routledge Handbook of Applied Communication*, eds Frey L. Cissna K., (New York, NY: Routledge), 355–379.

[B26] Englert B. Helmig B. (2018). Volunteer performance in the light of organizational success: a systematic literature review. *Voluntas* 29 1–28. 10.1007/s11266-017-9889-2

[B27] Etzioni A. (1973). The third sector and domestic mission. *Public Admin. Rev.* 33 314–323. 10.2307/975110

[B28] Farmer S. M. Fedor D. B. (2001). Changing the focus on volunteering: an investigation of volunteers’ multiple contributions to a charitable organization. *J. Manag.* 27 191–211. 10.1177/014920630102700204

[B29] Festinger L. (1954). A theory of social comparison processes. *Hum. Relat.* 7 117–140. 10.1177/001872675400700202

[B30] Friese S. (2013). *ATLAS.ti 7: User Guide and Reference.* Berlin: Scientific Software Development GmbH.

[B31] Ganesh S. McAllum K. (2012). Volunteering and professionalization: trends in tension. *Manag. Commun. Q.* 26 152–158. 10.1177/0893318911423762

[B32] Henriksen L. S. Rathgeb Smith S. Zimmer A. (2012). At the eve of convergence? Transformations of social service provision in Denmark, Germany, and the United States. *Voluntas* 23 458–501. 10.1007/s11266-011-9221-5

[B33] Hwang H. Suárez D. (2019). “Beyond service provision: advocacy and the construction of nonprofits as organizational actors,” in *Agents, Actors, Actorhood: Institutional Perspectives on the Nature of Agency, Action, and Authority (Research in the Sociology of Organizations)*, Vol. 58 eds Hwang H. Colyvas J. Drori G., (Bingley: Emerald Publishing Limited), 87–109. 10.1108/s0733-558x20190000058007

[B34] Jehn K. (1995). A multimethod examination of the benefits and detriments of intragroup conflicts. *Adm. Sci. Q.* 40 256–282. 10.2307/2393638

[B35] Jehn K. (1997). A qualitative analysis of conflict types and dimensions in organization groups. *Admin. Sci. Q.* 42 530–557. 10.2307/2393737

[B36] Jehn K. Mannix E. A. (2001). The dynamic nature of conflict: a longitudinal study of intragroup conflict and group performance. *Acad. Manag. J.* 44 236–251. 10.2307/3069453

[B37] Kamerāde D. (2015). Third Sector impacts on human resources and community. *Paper Presented at the* TSI Working Paper No. 3 Working Paper Series No.134. Seventh Framework Programme (grant agreement 613034), European Union, (Brussels: Third Sector Impact).

[B38] King D. (2017). Becoming business-like: governing the nonprofit professional. *Nonprofit Volunt. Sect. Q.* 46 241–260. 10.1177/0899764016663321

[B39] King N. (2004). “Using templates in the thematic analysis of text,” in *Essential Guide to Qualitative Methods in Organizational Research*, eds Cassell C. Symon G., (London: Sage).

[B40] Kreutzer K. Jäger U. (2011). Volunteering versus managerialism: conflict over organizational identity in voluntary associations. *Nonprofit Volunt. Sect. Q.* 40 634–661. 10.1177/0899764010369386

[B41] Kumar N. Stern L. W. Anderson J. C. (1993). Conducting interorganizational research using key informants. *Acad. Manag. J.* 36 1633–1651. 10.5465/256824

[B42] Macduff N. (2012). “Volunteer and staff relations,” in *The Volunteer Management Handbook: Leadership Strategies for Success*, 2nd Edn, ed. Connors T. D., (Hoboken, NJ: John Wiley & Sons, Inc).

[B43] Maier F. Meyer M. Steinbereithner M. (2016). Non-profit organizations becoming business-like: a systematic review. *Nonprofit Volunt. Sect. Q.* 45 64–86. 10.1177/0899764014561796

[B44] McAllum K. (2018). Volunteers as boundary workers: negotiating tensions between volunteerism and professionalism in nonprofit organizations. *Manag. Commun. Q.* 32 534–564. 10.1177/0893318918792094

[B45] Medina F. J. Munduate L. Dorado M. A. Martínez I. Guerra J. M. (2005). Types of intragroup conflict and affective reactions. *J. Manager. Psychol.* 20 219–230. 10.1108/02683940510589019

[B46] Medina F. J. Munduate L. Guerra J. (2008). Power and conflict in cooperative and competitive contexts. *Eur. J. Work Org. Psychol.* 17 349–362. 10.1080/13594320701761790

[B47] Mook L. Farrell E. Chum A. Handy F. Schugurensky D. Quarter J. (2014). Individual and organizational factors in the interchangeability of paid staff and volunteers: perspectives of volunteers. *Can. J. Nonprofit Soc. Econ. Res.* 5 65–85.

[B48] Müller-Stewens G. Dinh T. Hartmann B. Eppler M. J. Bünzli F. (2019). *The Professionalization of Humanitarian Organizations. The Art of Balancing Multiple Stakeholder Interests at the ICRC.* Switzerland: Springer Ebook.

[B49] Munduate L. Medina F. J. (2017). “How does power affect those who have it and those who don’t?,” in *An Introduction to Work and Organizational Psychology*, eds Chmiel N. Fraccaroli F. Sverke M., (Chichester: Wiley), 176–191. 10.1002/9781119168058.ch10

[B50] Nesbit R. Christensen R. K. Brudney J. L. (2017). The limits and possibilities of volunteering: a framework for explaining the scope of volunteer involvement in public and nonprofit organizations. *Public Admin. Rev.* 78 502–513. 10.1111/puar.12894

[B51] Netting F. E. Nelson W. Borders K. Huber R. (2008). Volunteers and paid staff relationships. *Admin. Soc. Work* 28 69–89. 10.1300/j147v28n03_04

[B52] Pearce J. (1993). *Volunteers: The Organizational Behavior of Unpaid Workers.* London: Routledge.

[B53] Penner L. A. Dovidio J. E. Piliavin J. A. Schroeder D. A. (2005). Prosocial behavior: multilevel perspectives. *Annu. Rev. Psychol.* 56 365–392. 10.1146/annurev.psych.56.091103.070141 15709940

[B54] Piñón J. (2010). *Invisibles, Precarios y Solidarios: Lo Que El Género Desvela: Empleo y Trabajo Voluntario en Organizaciones de Intervención Social y De Cooperación al Desarrollo.* Doctoral Dissertation, Universidad Complutense de Madrid, Madrid.

[B55] Rimes H. Nesbit R. Christensen R. K. Brudney J. L. (2017). Exploring the dynamics of Volunteer and staff interactions. *Nonprofit Manag. Leadersh.* 28 195–213. 10.1002/nml.21277

[B56] Salamon L. M. (2010). Putting the civil society sector on the economic map of the world. *Ann. Public Coop. Econ.* 81 167–210. 10.1111/j.1467-8292.2010.00409.x

[B57] Salamon L. M. (2015). Introduction: the nonprofitization of the welfare state. *Voluntas* 26 2147–2154. 10.1007/s11266-015-9638-3

[B58] Salomon L. M. Anheier H. K. (1997). The third world’s third sector in comparative respective. *Paper Presented at Working papers of The Johns Hopkins Comparative Nonprofit Sector Project, no. 24*, (Baltimore, MD: The Johns Hopkins Institute for Policy Studies).

[B59] Studer S. (2016). Volunteer management: responding to the uniqueness of volunteers. *Nonprofit Volunt. Sect. Q.* 45 668–714. 10.1177/0899764015597786

[B60] Vantilborg T. (2015). Volunteers’ reactions to psychological contract fulfillment in terms of exit, voice, loyalty, and neglect behavior. *Voluntas* 26 604–628. 10.1080/09585190801995849

[B61] Will M. G. Roth S. Valentinov V. (2018). From nonprofit diversity to organizational multifunctionality: a system – theoretical proposal. *Admin. Soc.* 50 1015–1036. 10.1177/0095399717728093

[B62] Wilson J. (2000). Volunteering. *Annu. Rev. Sociol.* 26 215–240. 10.1146/annurev.soc.26.1.215

